# Machine learning and dose response effect models: an integrated approach to analyze the association between environmental variables and Cuneo Emergency Department admissions for Acute Otitis Media (2007-2015)

**DOI:** 10.1007/s00484-026-03226-0

**Published:** 2026-05-19

**Authors:** M Rondinone, V Condemi, M Gestro, V Telesca

**Affiliations:** 1 Department of Engineering, University of Basilicata, Potenza, Italy; 2 Department of Biomedical Sciences for Health, University of Milan, Milan, Italy

**Keywords:** Acute Otitis Media, Emergency Department data, Machine Learning, Dose response models, Biometeorology, Air pollution

## Abstract

Acute otitis media (AOM) is a leading cause of pediatric Emergency Department visits, particularly among children under five years of age. Although its seasonal pattern is well established, the role of air pollution and meteorological factors remains unclear. This study aims to investigate their impact on daily AOM visits by integrating machine learning and epidemiological approaches. We conducted a retrospective analysis of pediatric AOM diagnoses (2007–2015) at S. Croce and Carle Hospital (Cuneo, Italy). Predictors included PM10, NO₂, O₃, and eleven meteorological variables. Ensemble machine learning models (Random Forest, XGBoost, and AdaBoost) were trained and validated using 10-fold cross-validation. Model interpretability was assessed through SHAP values. Distributed Lag Nonlinear Models (DLNM) were applied to estimate delayed exposure–response relationships over lag periods of 0–1, 0–3, 0–5, and 0–10 days, with results expressed as Relative Risks (RRs) and 95% Confidence Intervals (CIs). AdaBoost showed the best performance (R² = 0.974; MAE = 0.019 cases/day; cross-validated R² = 0.987). SHAP analysis identified mean temperature as the most influential predictor (44%), while PM10, NO₂, and O₃ each contributed approximately 10%. DLNM analysis confirmed a strong and consistent effect of temperature across all lag periods (RR > 1.20, CI > 1). Moderate associations were observed for NO₂ and PM10 (RR: 1.02–1.04). O₃ exhibited smaller but significant effects at shorter lags (RR = 1.01 at 0–1 days; RR = 1.02 at 0–3 days; CI > 1). Environmental factors, particularly temperature, play a significant role in pediatric AOM incidence. The integration of machine learning and DLNM enhances predictive accuracy and improves the understanding of exposure timing. These findings support the development of early warning systems and targeted preventive strategies under adverse environmental conditions. Further validation in larger urban settings is needed.

## Introduction

Acute otitis media (AOM) is among the most common diseases in children and adolescents, imposing a considerable economic burden on healthcare systems due to emergency department (ED) visits, unscheduled medical examinations, and antibiotic prescriptions (Schilder et al. 2016; Hoffman et al. 2013; Monasta et al. 2012; Rovers et al. 2004). A global analysis estimated a mean incidence of 10.8 cases per 100 individuals per year, with over half (51%) occurring in children under five years of age. Incidence peaks in the first year of life (45.3 per 100), remaining highest in the 1–4-year age group (61 per 100) (Monasta et al. 2012).

After upper respiratory infections, AOM is the second most frequent diagnosis in children visiting EDs (Berman 1995; Rovers et al. 2004). Acute otitis media (AOM) is the second most frequent diagnosis for children in the Emergency Department, trailing only upper respiratory infections (Berman 1995; Rovers et al. 2004). Given its high prevalence in the pediatric population, AOM represents a significant health concern. It can be associated with serious complications such as acute mastoiditis, which is particularly common in the first years of life (Bridwell et al. 2024; Groth et al. 2012). Furthermore, recurrent episodes can lead to hearing loss, potentially resulting in delayed language development and subsequent behavioral, cognitive, or developmental difficulties in both children and adolescents.

The onset of middle ear inflammation is influenced by genetic, anatomical, infectious, and environmental factors. Previous studies have linked AOM to sociodemographic, ethnic, and genetic susceptibility (Casselbrant et al. 2009; McCormick et al. 2011; Vakharia et al. 2010; Lasisi et al. 2007). Children with craniofacial anomalies, such as cleft palate, are at particular risk (Sheahan et al. 2003).

Meteorological conditions also play a role. Low winter temperatures and seasonal respiratory infections significantly increase AOM risk (Sprem et al. 1993; Bluestone et al. 2007; Gestro et al. 2017; Tian et al. 2020). Upper respiratory infections (URIs), common in cold seasons, are the most frequent comorbidity, with AOM incidence reaching 40–70% in association with viral upper airway infections (Winther et al. 2007; Chonmaitree et al. 2008; Mandel et al. 2008).

Air pollution has also been identified as a contributing factor to AOM. This association has been supported by several studies, including an Italian multicenter study (Scarinzi et al. 2013) which examined the relationship between air pollution and urgent hospital admissions in 25 Italian cities. The authors reported significant short-term effects of PM10, PM2.5, and NO2 on hospitalizations for respiratory diseases, particularly during the cold season and among the pediatric population (0–14 years). Similarly, traffic-related air pollution was investigated by Macintyre et al. 2014), showing a specific association with NO2 (OR = 1.09; 95% CI 1.02–1.16). A systematic review by Bowatte et al. (2018) further confirmed that NO2 shows the most consistent association with Otitis Media (OM) in children across various epidemiological studies. Finally, Park et al. (2018) and Wenhui et al (2024) reported a higher incidence of AOM in children under 15 years associated with NO2 and O3 concentrations exceeding reference values.

This study tests the hypothesis that AOM-related ED visits in Cuneo are influenced by meteorological and air pollution covariates, aiming to support prevention strategies targeting modifiable risk factors such as cold exposure and air pollution. An integrated approach combining Machine Learning (ML) and Distributed Lag Non-linear Models (DLNMs) (Gasparrini 2014) is proposed, enabling a more robust estimation of these relationships while accounting for linearity, non-linearity, and temporal dependencies.

The use of ML in otology has recently expanded. ML has been applied to AOM diagnosis (Habib et al. 2022; Esposito et al. 2021; Ding et al. 2023; Ezzibdeh et al. 2022; Rapoport et al. 2024) and to other otologic conditions, improving diagnostic accuracy and efficiency (Pizzulli et al. 2021; Castronuovo et al. 2023; Telesca et al. 2023). Reviews highlight ML’s role in automating otitis diagnosis and its potential across otology, from hearing aid optimization to vestibular disorders and image-based assessments (Ngombu et al. 2023; You et al. 2020).

Recent studies have also explored immunological and microbiome-related factors. Recurrent AOM may indicate Inborn Errors of Immunity (IEI), warranting immunological screening (Bardou et al. 2023). Conversely, modulation of the gut microbiome—particularly with probiotics— has shown promise in improving clinical outcomes (Godur et al. 2023).

## Methods

The present study is a retrospective observational study design, based on the Emergency Department (ED) admissions database of S. Croce Hospital (Cuneo, Italy) from 2007 to 2015 (a total of 1826 days). The variables collected in the dataset included: age, sex, ED admission date (including time, day, month, and year of access), triage code, nationality, municipality of residence, and mode of hospital discharge. Patients with AOM access were identified based on the following ICD-9 codes: 382.9, 381.00, 381.01, 381.02, 382.00, 383.00 as the primary diagnosis, and as a secondary diagnosis when confirmed by medical history and physical examination, in the presence of clinically related conditions as the primary diagnosis. ICD-9 codes are standardized diagnostic classifications used to define specific clinical conditions and, in this study, correspond respectively to: 382.9 (unspecified otitis media), 381.00 (acute nonsuppurative otitis media, unspecified), 381.01 (acute serous otitis media), 381.02 (acute mucoid otitis media), 382.00 (acute suppurative otitis media without spontaneous rupture of the tympanic membrane), and 383.00 (acute mastoiditis without complications). We excluded, for quality control purposes, AOM patients with: impaired immune systems, neoplastic rhino-pharyngeal diseases, cleft palate or otitis barotrauma; patients with a second ED visit within the 5 days of the first visit; and individuals residing in municipalities with an altitude > 1,000 m, due to differences in air pollutant concentrations and meteorological characteristics of the geographical context analyzed (Gestro et al. 2017). The ED database is entirely anonymized, according to the privacy code: Regulation (EU) 2016/679 (General Data Protection Regulation, GDPR); it is a completely de-identified dataset and, as such, did not require ethics committee approval. No patient contact was made, and patients could not be traced.

### Meteorological and air pollutant databases

**Fig. 1:**
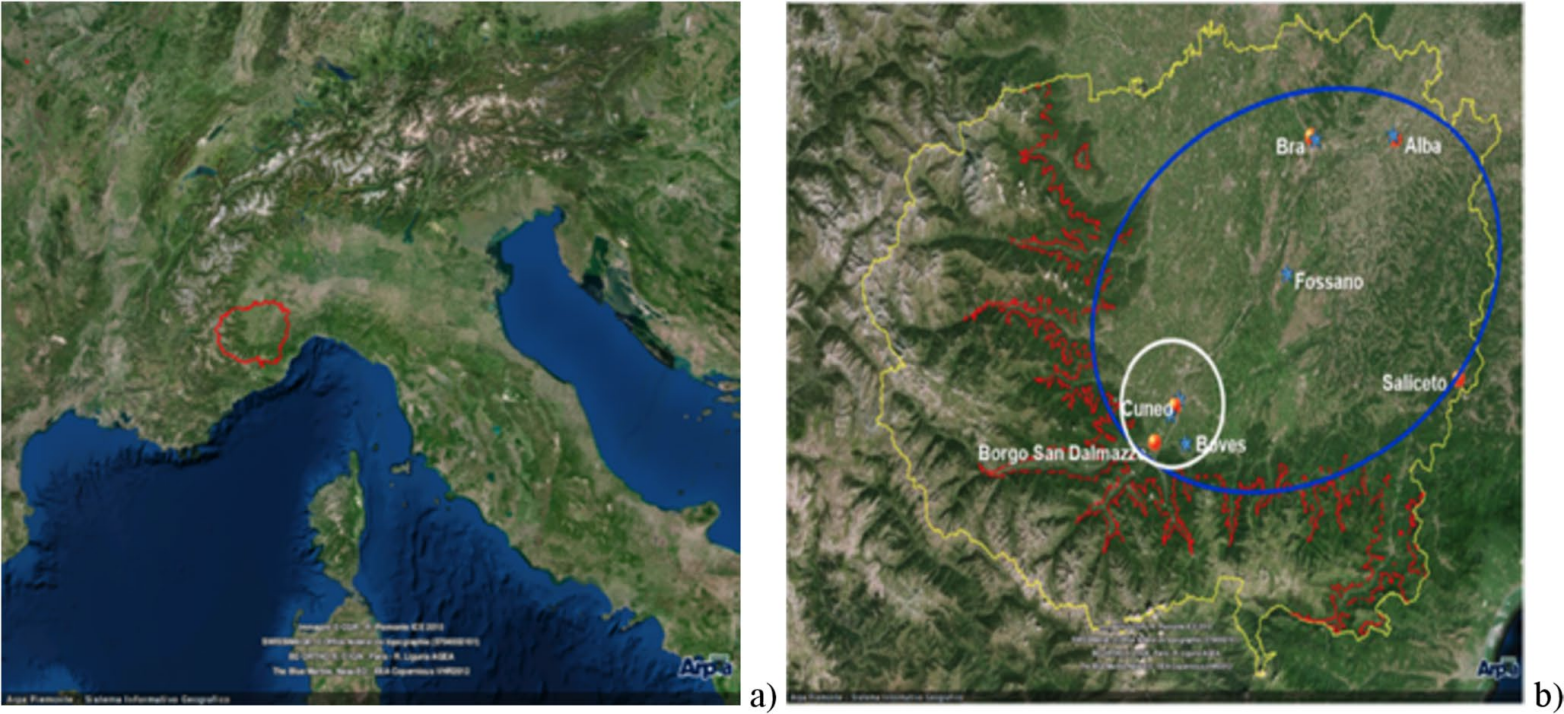
Geographical context of Cuneo and its province (**a**) National scale view (**b**) Local scale view including province border (yellow line) isoaltimetric line (red line) study area (blue line) and conurbation of Cuneo (white line) Sampling sites meteorological sites (blue stars) and air pollutant sites (yellow circles)

The meteorological and air pollutant databases of the Province of Cuneo for the period 2007–2015 were provided by ARPA Piedmont. Meteorological variables included: temperature (T_avg_, T_max_, and T_min_), Relative Humidity (RH_avg_, RH_max_, and RH_min_), Global radiation (Rad), Atmospheric pressure (P_mean_), Dewpoint (DP_mean_), Average and Maximum gust of wind (W_avg_ and W_max_). Air pollutants included: PM₁₀, NO₂, O₃. Daily averages were calculated for all meteorological variables. Meteorological and air pollutants data were analyzed using standard descriptive statistics. To improve control of the geographic variability, we used data from two reference stations selected for meteorological variables (Cuneo Cascina Vecchia) and air pollutants (Cuneo Alpini). Additionally, three supplementary monitoring stations for each reference site were included for comparison. From a climatic point of view, the Province of Cuneo, characterized by hilly areas and high plains, is classified according to the Köppen system as a mesothermal climate (C group), typical of mid-latitude temperate regions. The geographical context of the province and the specific area under study are shown in two maps (Fig. 1a–b). Cuneo is the third largest Province in northwestern Italy (Po Valley), with 592,303 residents. The population of Cuneo and its nine neighboring municipalities is 120,070 (all demographic data refer to 2015). The average altitude, calculated across municipalities, is 558.1 m.

### Data processing and statistical -machine learning methods

**Fig. 2:**
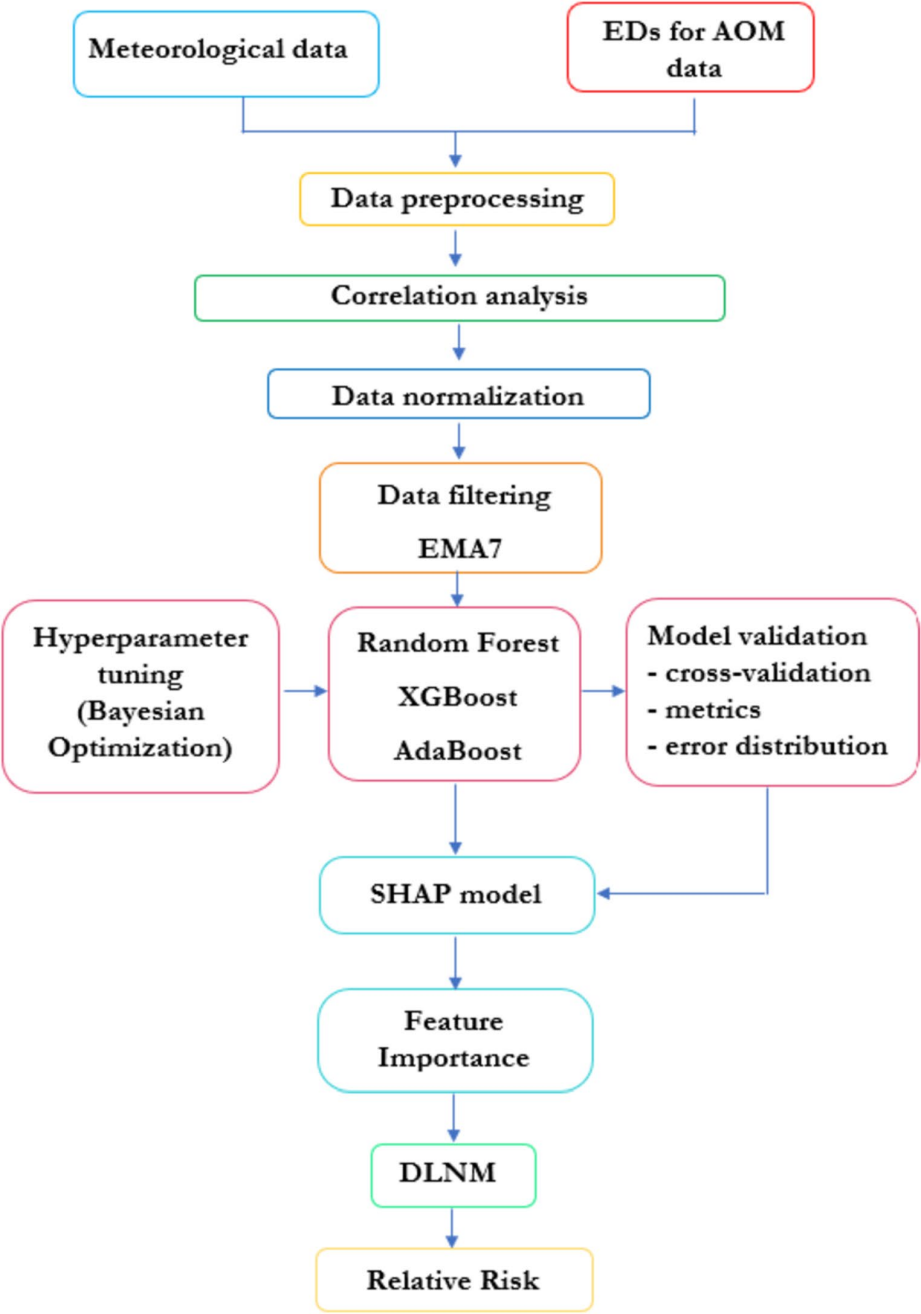
Methodological flow chart

Hoffman et al. 2013 indicated the need to improve statistical methods of analysis that account for complexity of data and enhance the knowledge of risk factors of AOM. In order to investigate the complex web of associations for AOM, we propose a statistical modeling framework structured into two stages and multiple steps within each stage. The study aims to identify the meteorological and air pollutant covariates that have the highest impact on Ed visits for AOM disease. To achieve this, a multi Stage-step approach was adopted, as shown in Fig. 2. The first step involved analyzing correlations between features and outcome variables. This was achieved by calculating Pearson’s correlation coefficient, r, and the p-value. Pearson’s coefficient measures the strength of the linear relationship between two variables, while the p-value evaluates the statistical significance of the correlation. Subsequently, the data were normalized and smoothed using a 7-day exponential moving average (EMA7) to regularize them. This technique is widely used in time series analysis to reduce short-term fluctuations and highlight underlying temporal patterns, allowing a more stable representation of the data and improving the ability of the models to capture consistent trends over time. To address the problem, an ensemble modeling approach was adopted, which combines different machine learning models to improve predictive performance. Specifically, three machine learning algorithms were compared:

- **Random Forest**: A supervised learning algorithm that employs multiple decision trees to make predictions. Each tree is built on a random subset of the dataset, and final predictions are obtained by averaging or voting among the trees, reducing overfitting.
- **XGBoost**: An advanced boosting algorithm that optimizes residual errors by constructing sequential decision trees, where each subsequent tree corrects the errors of the previous ones. It is known for its speed and efficiency.
- **AdaBoost**: A boosting algorithm that combines weak learners (typically simple decision trees) into a strong model. Each new model is trained to correct the errors of previous models by progressively increasing the weight of misclassified instances.

The models were trained using 70% of the dataset and tested on the remaining 30%, with subsets obtained through random sampling. To further ensure the robustness and generalizability of the results, a 10-fold cross-validation procedure was also applied, allowing model performance to be evaluated across multiple data partitions and reducing the risk of overfitting. The three models were optimized through hyperparameter tuning using Bayesian optimization, a technique that employs a probabilistic model (typically a Gaussian process) to predict performance across different hyperparameter combinations, reducing the number of required experiments and intelligently exploring the hyperparameter space. The performance of the three models was evaluated using the Mean Absolute Error (MAE), the coefficient of determination (R²), and the distribution of errors between the model-predicted and actual values. Additional error metrics (MSE, RMSE, and MAPE) were also computed to provide a more comprehensive evaluation of model accuracy. A 10-fold cross-validation procedure was additionally applied to ensure that performance metrics remained stable across different samples.

Then, using the SHAP method, a feature importance analysis based on Explainable Artificial Intelligence (XAI) was conducted to determine the most significant variables and their ranking. This approach measures the contribution of each feature to the model’s predictions by computing Shapley values (Zhang et al. 2020). Based on game theory, SHAP provides a consistent and locally faithful interpretation of the influence of each variable in the model’s decision-making process (Mangalathu et al. 2020; Nohara et al. 2022). This method enhances model interpretability by quantifying the relative contribution of each predictor to the final output.

Finally, a Distributed Lag Non-Linear Model (DLNM) is applied to estimate the Relative Risk (RR) of AOM admissions associated with the most significant meteorological and air pollutant variables identified in the previous steps. The DLNM model is a statistical model that allows the estimation of the linear and non-linear delayed effects of meteorological and air pollutant variables on ED admissions for AOM. Reference points (centered values) for the DLNM were chosen based on epidemiological interpretability: the mean was used for temperature and the first quartile (Q1) for air pollutants. By following this methodology, the study aims to identify the variables that have the highest impact on ED admissions for AOM disease. This can help inform strategies for reducing ED admissions for AOM by addressing the variables that have the most significant impact. The statistical analyses and Machine Learning/EMA7 pipeline were developed using the Python environment and R Statistical Software version 4.2.0. All models were evaluated in both training and testing phases, and the consistency of results was verified through cross-validation procedures.

### Seasonal and temporal autocorrelation analysis

The association between day of year and outcome was analyzed using quasi-Poisson regression models with natural spline functions for long-term trends, while adjusting for day of the week and holidays as covariates.

To account for serial dependence, autoregressive models of increasing order, AR(Bardou et al. 2023)–AR(Bridwell et al. 2024), were estimated, and residual autocorrelation was evaluated using the Ljung–Box Q test. This procedure allowed assessment of remaining temporal dependence not captured by the primary model specification and ensured appropriate handling of seasonal and autocorrelated structures in the time series.

## Results

### Descriptive statistics

Table 1 provides descriptive statistics on the daily time series of environmental variables (meteorological and air pollutants) and the number of cases admitted in the ED. The total number of ED visits is 695,675 with daily average of 211 and an average age of 42.52 years. The number of ED visits was 226,343 (2007–2009), 234,408 (2010–2012) and 234,924 (2013–2015). Before application of exclusion criteria, 5,924 ED visits for AOM were isolated, with a daily average of 2.34 and annual of 658.2 with 3,227 males (54,5%) and 2,697 females (45,5%).

**Table 1:** Statistical summary of outdoor meteorological variables, air pollutant covariates, and all ED visits (2007–2015)

Variables	Units	Min	1st Qu.	Median	Mean	3rd Qu.	Max	SD
**Tavg (°C)**	3287	–8.6	5.4	12.5	12.3	19.1	28.2	7.9
**RHavg (%)**	3287	26	57	69	68	82	100	16.6
**P(hP)**	3287	969	1011	1016	1016	1020	1040	7.7
**Rad (W/m²)**	3287	0.2	7.1	12.8	13.71	20.4	32.4	8.1
**Windavg (Km/h)**	3287	0	1.3	1.6	1.5	1.9	3.5	0.5
**O**_**3**_ **(µg/m**^**3**^**)**	3287	3	38	61	62	81	149	27.4
**PM**_**10**_ **(µg/m**^**3**^**)**	3287	1	14	22	26	33	121	17.6
**NO**_**2**_ **(µg/m**^**3**^**)**	3287	8	21	29	31.1	39	86	12.6
**AOM ED admissions**	3287	0	0	1	1653	2	10	1.6

After application of exclusion criteria, the daily number of ED visits observed was 4,534 including 2,489 females and 2,945 males, average age 15.02 and SD ± 19.21 for females (45.84%), 14.01 ± 18.61 for males (54.16%). The seasonal OM distribution for the entire period shows 1,724 ED visits in winter (31.72%), 1,526 (28,08%) in spring, 941 (17.31%) in summer and 1,243 (22.87%) in autumn. Time series analysis confirmed the presence of a marked seasonal pattern in the outcome. Evaluation of residual autocorrelation using the Ljung–Box Q test indicated significant serial dependence in the AR(Bardou et al. 2023)–AR(Biggeri et al. 2001) models, whereas the AR(Bluestone and Klein 2001) model was the most parsimonious specification able to eliminate residual autocorrelation (*p* = 0.63). This final model was therefore used to estimate seasonal effects more reliably.

The analysis of the Day Of Year (DOY) further highlighted a clear seasonal structure in ED admissions for AOM. The risk curve showed increased incidence during colder months, a reduction in summer, and a subsequent rise in late autumn. Relative risk estimates across DOY quantiles confirmed this pattern: the highest risk was observed at the 10th percentile (day 51, RR = 1.46; 95% CI: 1.30–1.64), followed by persistently elevated values at the 25th percentile (day 110, RR = 1.32; 95% CI: 1.17–1.49). A reduction was observed during late summer (75th percentile, day 293, RR = 1.17; 95% CI: 1.05–1.31), with a subsequent increase toward the end of the year (90th percentile, day 325, RR = 1.24; 95% CI: 1.10–1.40).

### ML models

Following the methodological framework, the first step of the analysis involved studying the correlations between the independent variables (features) and the target variable. This was achieved by calculating the Pearson correlation coefficient, r, and the associated p-value.

**Fig. 3:**
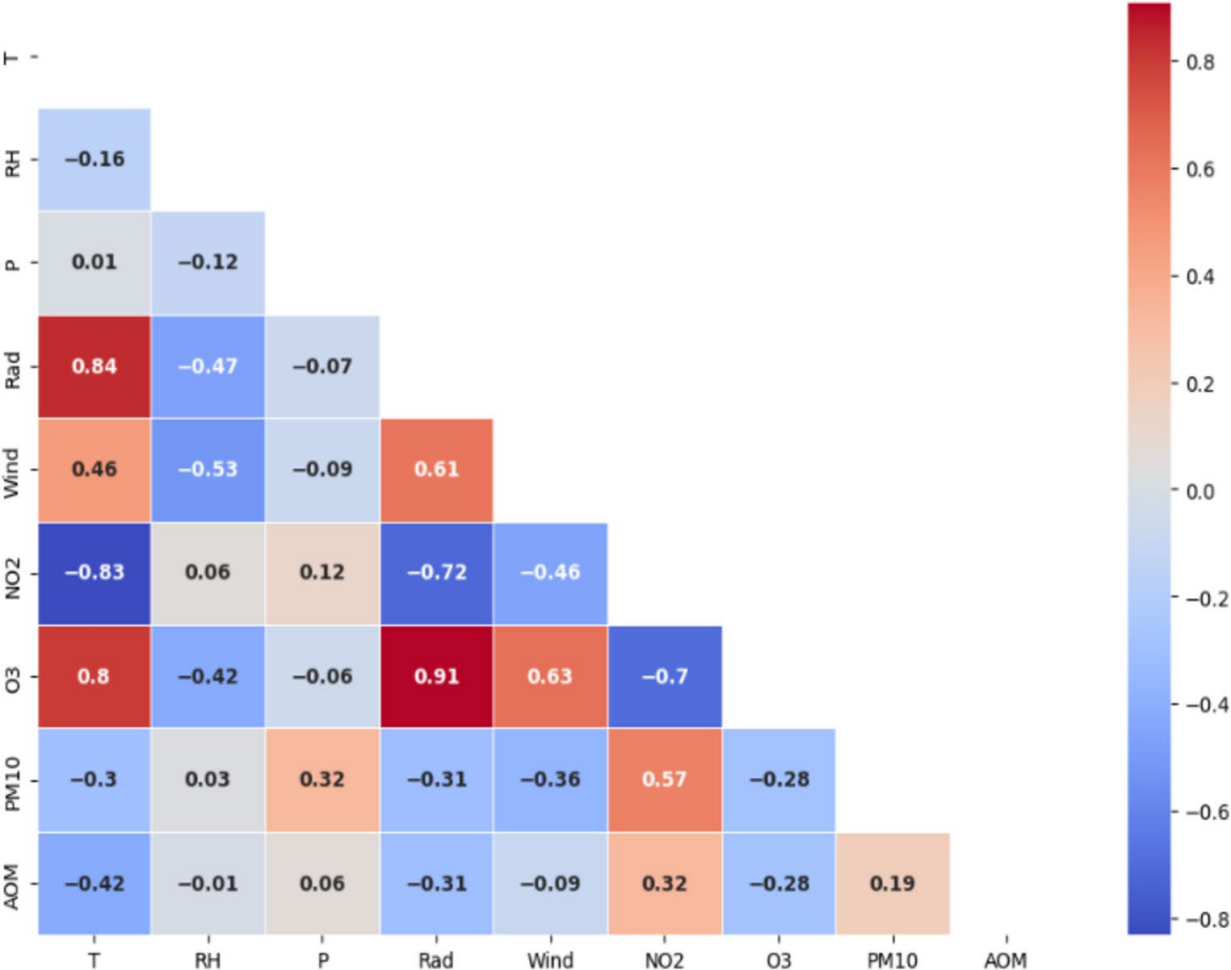
Correlation matrix

However, the mere presence of a high value of r is not sufficient to determine whether the correlation is statistically significant. For this reason, the p-value was also calculated in the modeling process, which measures the probability that the observed correlation is due to chance. A p-value below a common threshold (e.g., 0.05) indicates that the correlation is statistically significant, meaning it is unlikely to be due to chance; conversely, a high p-value suggests that the observed correlation might be the result of random variability in the data (Gogtay et al. 2017). In this case, the p-value remained below the threshold of 0.05, allowing us to conclude that the obtained correlation is statistically significant. In this regard, the correlation matrix is shown in Fig. 3.

The analysis of the correlation matrix allows for several observations. As can be seen, a negative correlation between AOM and temperature (*r* = − 0.42) emerges; this suggests that warmer climatic conditions could reduce the risk of AOM, although the strength of the correlation is not very high. A negative correlation is also observed with solar radiation (*r* = − 0.31), implying that an increase in solar radiation levels could be associated with a decrease in the number of otitis cases, although the correlation is moderate. A negative correlation is also found with O₃ (*r* = − 0.28), indicating that an increase in ozone concentration in the air could be associated with a slight reduction in the number of AOM cases. Positive correlations are found with NO₂ (*r* = 0.32) and PM_10_ (*r* = 0.19), suggesting that exposure to these pollutants could have a significant impact on increasing the risk of developing otitis.

After identifying the most relevant correlations, a data normalization process was applied to ensure that all variables were comparable and not influenced by different scales. Normalization helps to prevent variables with larger numerical values from dominating those with smaller values, ensuring a more balanced assessment of the relationships between variables.

Subsequently, to improve the quality of the analysis and reduce daily data variability, smoothing was applied through a 7-day Exponential Moving Average (EMA7). This step was crucial for stabilizing the data and preparing it for the application of predictive models, providing more stable inputs and reducing the risk of overfitting. Thus, this process ensures a more robust and reliable analysis of the relationships between variables, facilitating the interpretation of results and improving the quality of the subsequent modeling phases.

#### Training of machine learning models

Once the main correlations and data were defined, an ensemble model was implemented to optimize the prediction of AOM cases, leveraging the combination of multiple predictive algorithms. As previously mentioned, the models used were Random Forest, Extreme Gradient Boost, and Adaptive Boost. The three models were optimized using Bayesian optimization and their performances were subsequently compared (Table 2a).

**Table 2:** Descriptive statistics and model performance metrics (a) Ensemble model implementation and comparison of predictive algorithms (b) k-fold cross-validation results for XGBoost, Random Forest, and AdaBoost models. (a)

(a)		
Models Performance		
Random Forest	XGBoost	AdaBoost
R^2^ test (–) = 0.935	R^2^ test (–) = 0.927	R^2^ test (–) = 0.974
MAE test (case/day) = 0.091	MAE test (case/day) = 0.034	MAE test (case/day) = 0.019
(b)		
**Cross Validation (k fold = 10)**		
Random Forest	XGBoost	AdaBoost
R^2^ test (–) = 0.933	R^2^ test (–) = 0.944	R^2^ test (–) = 0.987
MAE test (case/day) = 0.087	MAE test (case/day) = 0.028	MAE test (case/day) = 0.019

The performance of the training/test phases of the three predictive model are assessed through some statistical indexes (Chicco et al. 2021), widely used in the literature: the determination coefficient (R^2^) and the Mean Absolute Error (MAE). Training a machine learning model using the Random Forest algorithm produced strong results, with the R² coefficient for the test set reaching 0.935. This indicates that the model demonstrates a high level of adaptability and accuracy when simulating a new dataset not used during training. The model’s performance was similarly validated by the Mean Absolute Error (MAE), which for the test set was 0.091 cases per day, indicating good predictive accuracy.

**Fig. 4:**
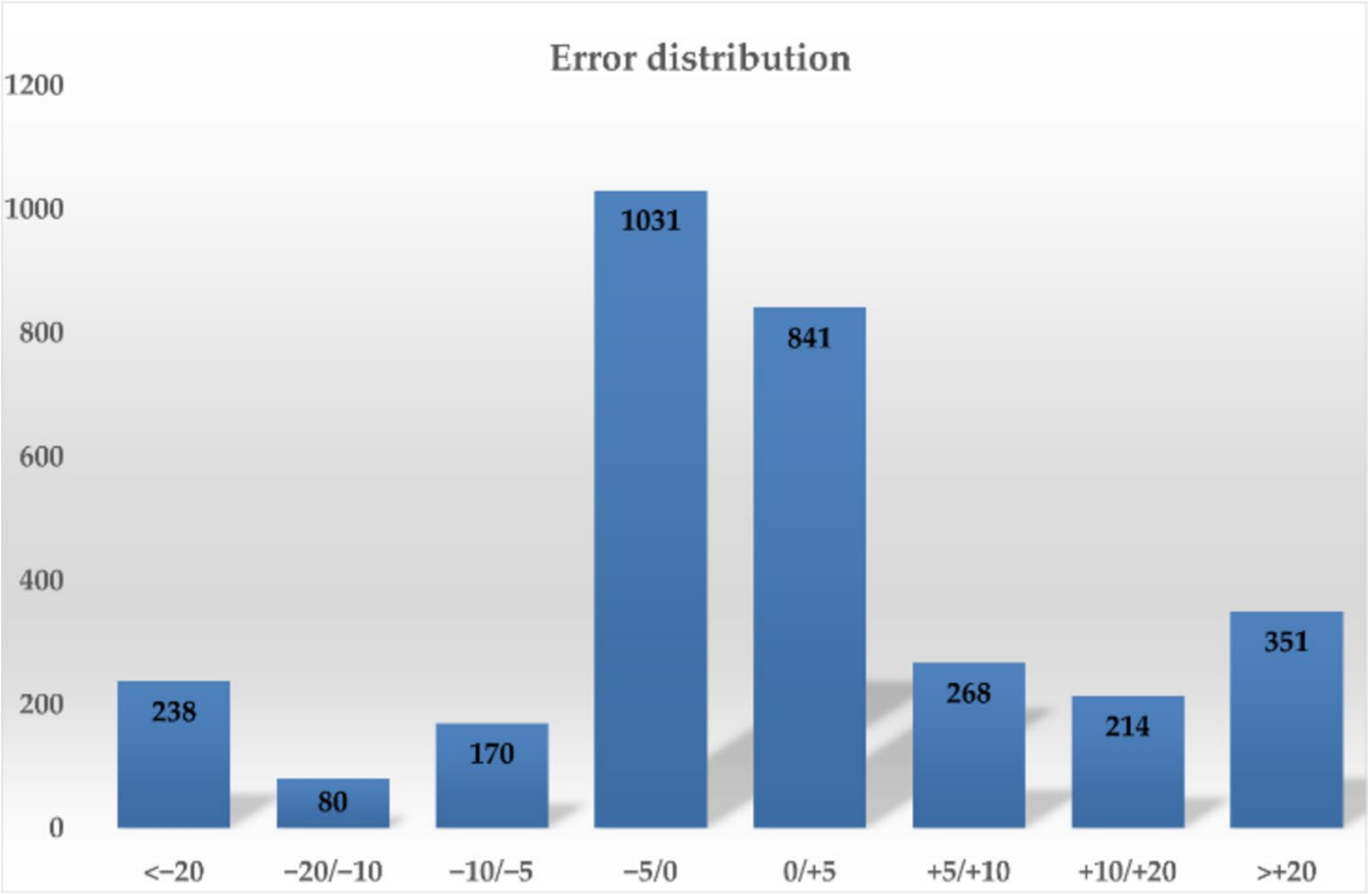
Error distribution of AdaBoost model results

In comparison, the XGBoost model showed competitive performance, with an R² of 0.927 and an MAE of 0.034 cases per day, outperforming Random Forest in precision. AdaBoost achieved the best results (R² = 0.974; MAE = 0.019), demonstrating high accuracy and precision. However, the choice of the best model was not based solely on these metrics, but also on the evaluation of error distribution (Fig. 4). AdaBoost, while exhibiting both overestimation and underestimation (errors within ≶ 20%), showed a more favorable error distribution (Fig. 4), a crucial element for robustness and generalization. Results indicate that 59% of simulated values fall within the ± 5% error range: 32.3% between − 5% and 0%, and 26.3% between 0% and + 5%. This highlights a strong adherence of the model to actual values, with minimal deviations within ± 5%. In conclusion, the analysis confirms the reliability of the AdaBoost-based simulation model in consistently reproducing observed values.

To further validate the robustness of the model and ensure its generalizability, a k-fold cross-validation procedure with k = 10 was applied (Table 2b). This technique involves splitting the dataset into 10 equally sized subsets (or “folds”). For each iteration, the model is trained on 9 of the 10 folds, and the remaining fold is used for testing (Nti et al. 2021). This process is repeated 10 times, each time using a different fold as the test set, ensuring that each observation is used for both training and testing.

The advantage of using k-fold cross-validation is that it provides a more comprehensive assessment of the model’s performance by evaluating it on different subsets of the data. This reduces the risk of overfitting, as the model is exposed to various training and testing combinations, allowing for a more accurate estimate of its performance on unseen data. Additionally, it helps in detecting any potential bias that could arise from a single split of the data, provides a more reliable estimate of model performance on unseen data. The average results of the k-fold cross-validation are provided in tabular format (Table 2b), where the model’s performance for each fold and the overall mean of the evaluation metrics are reported.

The results of the cross-validation are as follows:

- The R² coefficient for the test set indicates that the model explains 98.7% of the variance in the test data.
- The Mean Absolute Error (MAE) shows that, on average, the model’s predictions deviate by only 0.019 cases per day from the actual values.

These results confirm that the model performs reliably across different subsets of the data, demonstrating both strong predictive accuracy and generalizability. The low MAE value highlights the model’s ability to consistently provide accurate predictions on new, unseen data. These findings provide a solid foundation for applying the Feature Importance procedure, allowing us to identify the key variables that most influence the model’s performance in predicting Emergency Department visits for AOM.

#### SHAP feature importance

**Fig. 5:**
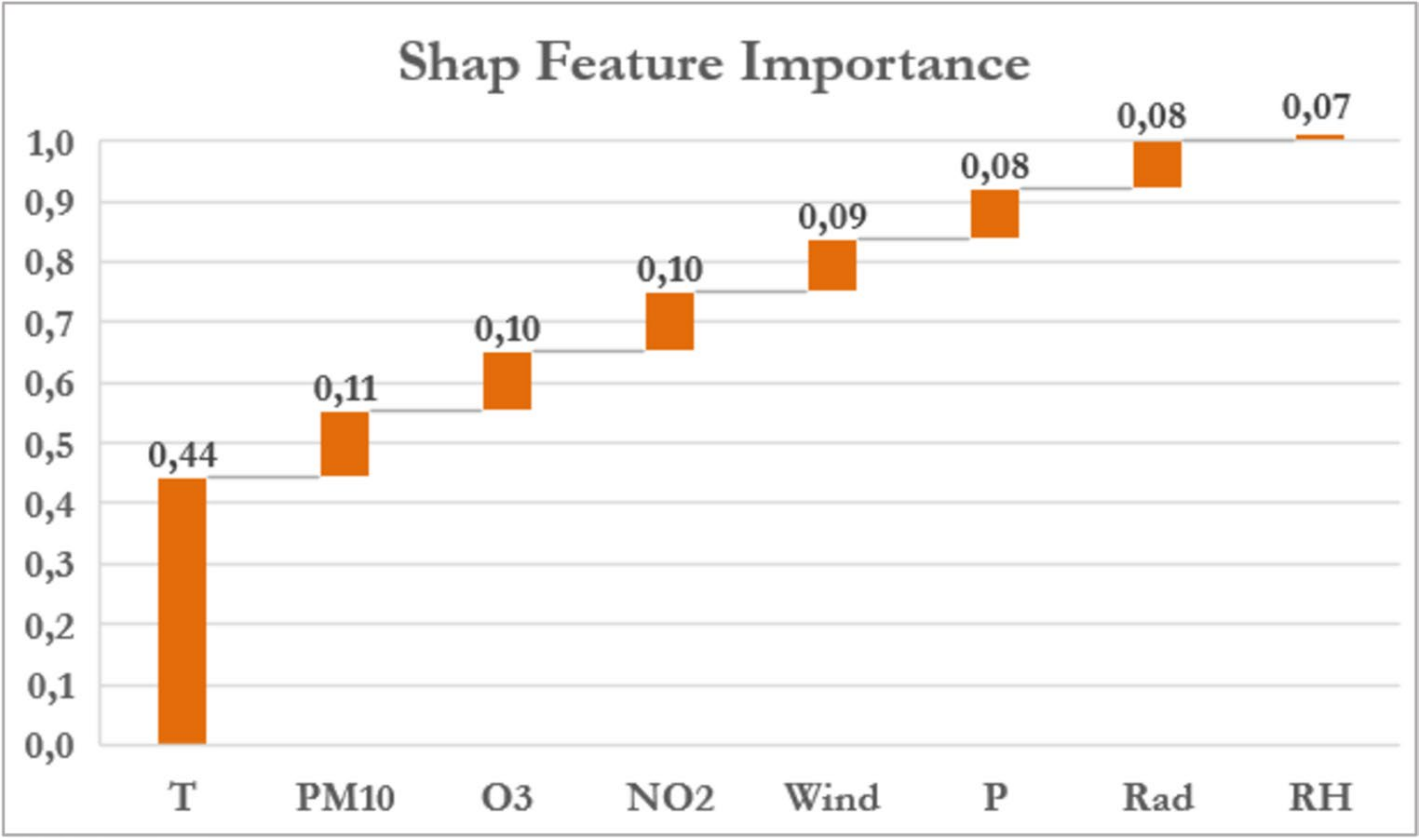
SHAP features importance analysis

The SHAP method (SHapley Additive exPlanations) is a widely used technique in the field of Explainable Artificial Intelligence (XAI) and is based on the principles of game theory (Barredo Arrieta et al. 2020; Shaikhina et al. 2021). This approach assigns a Shapley value to each independent variable in a model, the contribution of each feature to be quantified in relation to the prediction of the target variable. As shown in Fig. 5, the SHAP analysis provided a clear overview of the relative importance and behavior of the climatic and environmental factors considered. In particular, the bar chart shows that mean temperature is the variable with the greatest impact, contributing 44% to the model’s predictions. Since SHAP analysis assumes that the most relevant features are those whose combined contribution reaches at least 70–80%, the other important features in this case are PM_10_, O_3_ and nitrogen dioxide, whose contributions are very similar and therefore comparable in terms of importance.

### DLNM model

The outcome of Stage 2 (PGAMs combined with DLNM) is expressed in terms of RR (95% CI). As provided for by the statistical methodology and following the results expressed by the ML models T_avg_, NO_2_, O_3,_ PM_10,_ and solar radiation (Rad) were included separately in the DLNM routines. The parameters used for DLNM routines have been: Tavg: centered point at 12.5 (mean) and **±** 5 °C variation. NO₂: centered point at 25 µg/m³ (**±** 10 µg/m³ variation), PM10 with a centered point at 5 µg/m³ (**±** 10 µg/m³ variation), O₃: centered point at 20 µg/m³ (**±** 30 µg/m³ variation) and Rad with centered point at 5 W/m² (**±** 10 W/m² variation). The cross basis starting from lag-range 0–1 and subsequent ones up to lag-range 0–10 show for T_avg_ substantial effects across all lag ranges (0–1, 0–3, 0–7, and 0–10) with RR and 95% CI > 1. A similar but less important result was observed for NO_2_ and PM_10_ observed an RR and relative CI > 1 in the lag range from 0 to 1 to 0–10, in the shortest sequences it is always an RR and CI (RRlow and RRhigh) > 1. An identical situation has been observed for PM_10_ with a significant RR > 1 in all lag ranges. The O_3_ results are significant in the lag range 0–1 and 0–3 with RR and 95% CI > 1 across lag ranges from 0 to 1 to 0–10. The overall DLNM results of delayed effects (Quasi Poisson approach used for overdispersion) for T_avg_, NO_2_, O_3,_ PM_10,_ and Rad are shown in Table 3.

**Table 3:** Results for several DLNM routines - RR and CI for Tavg, NO_2_, O_3_, PM_10_, and Rad included in the model sequence and calendar factors (baseline approach). Response variable AOM

	Lag days	RR	95% CI low	95%CI high
T_avg_	0–1	1.206	1.171	1.242
	0–3	1.216	1.180	1.253
	0–5	1.227	1.191	1.265
	0–10	1.235	1.197	1.277
NO_2_	0–1	1.047	1.036	1.061
	0–3	1.074	1.059	1.088
	0–5	1.083	1.068	1.097
	0–10	1.085	1.070	1.101
PM_10_	0–1	1.021	1.011	1.032
	0–3	1.029	1.017	1.041
	0–5	1.033	1.020	1.047
	0–10	1.040	1.025	1.056
O_3_	0–1	1.019	1.004	1.035
	0–3	1.019	1.003	1.026
	0–5	1.016	0.999	1.033
	0–10	1.016	0.997	1.035
Rad	0–1	0.939	0.919	0.960
	0–3	0.932	0.911	0.954
	0–5	0.900	0.869	0.933
	0–10	0.910	0.887	0.934

## Discussion

The initial correlation analysis between meteorological and air pollution variables, and Acute Otitis Media (AOM) cases showed weak associations. Machine Learning models confirmed this trend, with modest performance (R^2^ = 0.515; MAE = 1.22 cases/day). Following verification of data stationarity (Dickey-Fuller test), the EMA7 model was applied to regularize the series. The new correlation analysis revealed: (i) negative correlations with average, maximum and minimum temperature, dew point humidity, temperature–humidity index, solar radiation, and ozone, suggesting that higher values are associated with a reduction in AOM cases; (ii) a positive correlation with NO₂, indicating higher levels may increase AOM; (iii) weak associations with maximum relative humidity, atmospheric pressure, and PM₁₀, suggesting limited influence.

Subsequent ML modeling showed strong performance: (i) R² = 0.974 on the test set, confirming robustness through cross-validation (further supporting model stability); (ii) MAE = 0.019 cases/day, indicating high accuracy; (iii) 59% of predictions within ± 5% error, with 32.3% between − 5% and 0% and 26.3% between 0% and + 5%. These extremely high predictive performances should be interpreted with caution. The application of the 7-day exponential moving average (EMA7) reduced short-term variability, making the data more regular and predictable. Therefore, a substantial part of the model’s predictive ability derives from smoothing and capturing temporal patterns rather than causal relationships between environmental exposures and outcomes. Results should be interpreted primarily in a predictive, not causal, framework. In addition, the very high performance achieved by the AdaBoost model raises the potential concern of overfitting. However, several elements support the generalization capability and controlled complexity of the model. Model performance was evaluated on an independent test set, providing an unbiased estimate of predictive accuracy. Furthermore, a 10-fold cross-validation procedure was performed, yielding highly consistent results (R² ≈ 0.987; MAE ≈ 0.019; see Table 2b), with limited variability across folds and consistently high performance across all folds. This stability indicates that the model performance is not dependent on a specific data partition and suggests robust generalization. Moreover, the complexity of the AdaBoost model was carefully controlled through hyperparameter selection, including the number of estimators (n_estimators = 715), learning rate (learning_rate = 1), and the choice of base estimator. These hyperparameters were optimized to balance bias and variance, effectively limiting the risk of overfitting while maintaining high predictive accuracy. The combination of cross-validation stability and optimized hyperparameters demonstrates that the model generalizes well to unseen data, reinforcing confidence in its predictive reliability. Overall, these findings support the reliability of the model despite its high predictive performance. AdaBoost, validated as the best-performing algorithm, was then used for SHAP-based Feature Importance analysis. The most influential variables were Tavg, PM₁₀, O₃, and NO₂. SHAP results showed that temperature strongly affects AOM at low values, while pollutant contributions vary by concentration (high PM₁₀, low O₃ and NO₂).

**Fig. 6:**
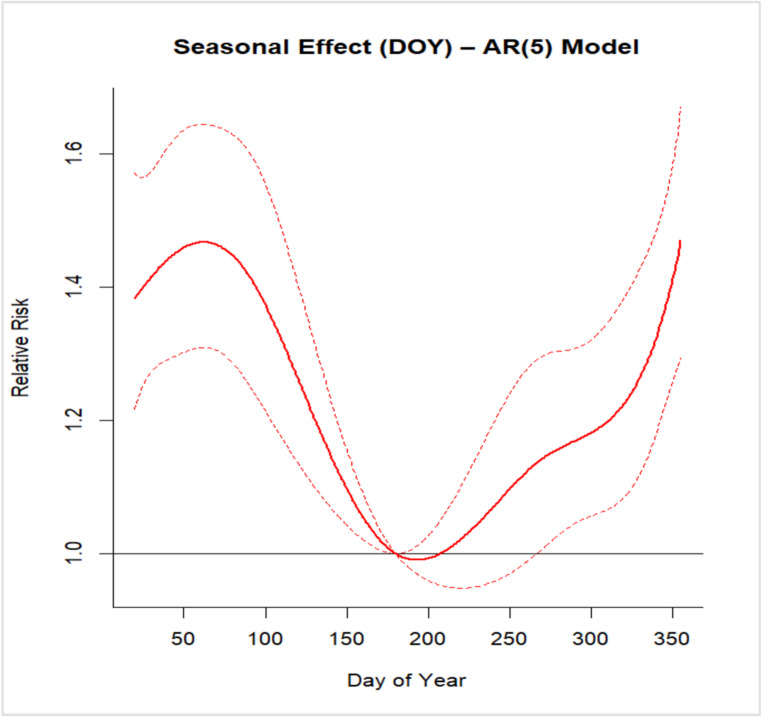
Seasonal effect – AR (Bluestone and Klein 2001) model

To contextualize these findings, a recent study by Mun and Chang (2022) was taken into consideration, as it developed predictive models for pediatric acute otitis media using both Poisson regression and advanced boosting-based machine learning techniques. Their results demonstrated that boosting methods more accurately predicted weekly AOM frequency compared to traditional regression, underlining the importance of environmental variables such as minimum temperature, daylight duration, and levels of NO₂ and O₃. Notably, they observed increased vulnerability to O₃ exposure in children under two years, highlighting the interaction between environmental factors and individual susceptibility. These findings are consistent with the present work, which also identifies temperature and air pollutants (NO₂ and O₃) as key drivers of AOM risk. Moreover, both studies applied SHAP methodology, enhancing model interpretability and reinforcing the validity of machine learning approaches for case prediction. Comparison with DLNM models showed overall agreement. Temporal structure analysis further confirmed the presence of a strong seasonal component in the outcome (Fig. 6). This was supported by the evaluation of autoregressive models and residual diagnostics using the Ljung–Box test, which indicated that lower-order models, AR(Bardou et al. 2023)–AR(Biggeri et al. 2001), did not fully capture serial dependence, whereas the AR(Bluestone and Klein 2001) specification adequately removed residual autocorrelation. These findings highlight that part of the observed variability in AOM admissions is driven by strong temporal dependence and seasonal patterns, which may influence both the estimated associations and the predictive performance of the models. In particular, the seasonal structure identified in the day-of-year analysis is consistent with the patterns observed in both ML and DLNM approaches, suggesting that temporal dynamics represent a key underlying driver of the observed effects. Average temperature emerged as the strongest predictor, representing ~ 40% of importance, exceeding each pollutant (10–11% each). Seasonal SHAP analysis highlighted wintertime low temperatures as the main risk factor. DLNM confirmed consistent effects of mean temperature across lag intervals (0–1, 0–3, 0–5, 0–10 days), with RR > 1.20, underscoring its role as a major determinant of AOM and aligning with previous studies (Nieratschker et al. 2023; Gestro et al. 2017; Xu et al. 2014). Preventive strategies are needed given risks of complications such as acute mastoiditis (Bridwell et al. 2024; Groth et al. 2012).

Pollutants also contributed, though less than temperature. SHAP and dose–response analyses identified NO₂ as a relevant factor, with RRs 1.04–1.08 across all lags, consistent with previous evidence (Bowatte et al. 2018; Park et al. 2018; Gestro et al. 2017; Xiao et al. 2016; Macintyre et al. 2014; Zemek et al. 2010; Kennedy et al. 2018). O₃ showed significant associations only at shorter lags (0–1, 0–3 days; RR 1.01–1.02), with weaker evidence at longer lags. This aligns with literature reporting mixed findings (Park et al. 2018; Tian et al. 2020). The seasonal mismatch between O₃ peaks (summer) and AOM prevalence (winter) may explain the weaker link. ML models nevertheless captured O₃ as a predictor due to its distinct seasonal peaks, despite fewer summer cases.

PM₁₀ was also confirmed as moderately associated with AOM across all lags (RR and CI > 1), in line with broader respiratory and cardiovascular literature and emerging but less consistent AOM-specific evidence (Park et al. 2018; Lee et al. 2020; Bowatte et al. 2018).

Other meteorological variables (wind, pressure, radiation, humidity) showed no notable associations in DLNM. Radiation was reported as an example due to strong collinearity with O₃ (correlation 0.91).

Overall, Tavg remains the dominant factor influencing AOM, followed by NO₂, O₃, and PM₁₀. These results align with prior studies (Scarinzi et al. 2003; Monasta et al. 2012; Hoffman et al. 2013; Xu et al. 2014; Macintyre et al. 2014; Gestro et al. 2017; Park et al. 2018; Kennedy et al. 2018; Lee et al. 2020). Pediatric populations are particularly vulnerable to low temperatures and, secondarily, to pollutants (NO₂, PM₁₀ in winter; O₃ in summer). Importantly, pollutant levels in Cuneo were lower than in other Italian urban areas (e.g., Turin), remaining below EU air quality limits (Directive 2008/50/EC) for NO₂ and PM₁₀ during 2007–2015. O₃, however, did not show significant declines, representing a persistent concern.

### Strength and limitations

The study’s strength is represented by the careful application of selection criteria and the detailed definition of the geographical sub-region in terms of both extent and altitude, thereby minimizing spatial variability of exposure.

Additionally, the integrated approach combining Machine Learning techniques and nonlinear statistical models (DLNM) enabled the development of a robust analytical framework capable of capturing both temporal patterns and complex relationships between environmental factors and AOM incidence. The use of a 7-day exponential moving average (EMA7) further enhanced model predictive performance by reducing short-term noise. Model performance was rigorously evaluated on an independent test set and through cross-validation, supporting the reliability and stability of predictive results within the dataset. However, the study also has several limitations:

i) The analysis is based on daily aggregated data (using the 7-day EMA), which improves model stability but reduces short-term variability, potentially affecting the precision of predictions for single-day fluctuations; ii) Environmental and meteorological data were obtained from fixed outdoor monitoring stations, without information on indoor exposure, which may lead to exposure misclassification and underestimate of individual-level variability;
iii) Strong seasonal patterns and temporal autocorrelation were observed in the outcome, as confirmed by autoregressive modeling and residual diagnostics. Although these structures were explicitly modelled (e.g., AR terms and spline-based seasonal components), residual temporal dependence may still influence both model performance and the estimation of associations, potentially leading to partial over- or under-estimation of effects in time-series analyses; iv) potential exposure misclassification arising from reliance on fixed outdoor monitoring stations, mitigated by detailed definition of the geographical sub-region in terms of both extent and altitude, excluding patients based on municipality of residence data. The integrated approach between Machine Learning techniques and nonlinear statistical models (DLNM) has allowed the development of a very robust framework. While the integration of these two techniques provides a more comprehensive understanding of environmental associations, the results should be interpreted as evidence of significant statistical relationships that support the development of early warning systems, rather than as biological proof of direct causality; v) the data analyzed in this study only reflect cases requiring ED utilization and do not account for AOM episodes managed in primary care settings; vi) the dataset pertains exclusively to the Province of Cuneo, which has relatively low pollution levels compared to larger urban areas, with pollutant concentrations generally lower than in major conurbations. Nevertheless, the methodological framework is robust and can be applied to other contexts, provided local data are available, although specific results may vary according to local conditions.

## Conclusions

The results of this study indicate that low temperatures, typical of the climatic context of Cuneo, play a fundamental role as an environmental risk factor associated with Emergency Department visit for AOM. This result is confirmed in previous publications, which indicate this factor as of primary importance. The pollutants examined (NO₂, O₃, PM_10_) show a more moderate impact, with varying significance, depending on the statistical model used. Considering the levels of air pollutants observed in Cuneo over the last twenty years, with constantly decreasing trends, further studies are needed, particularly in larger urban contexts, even in a multicenter mode, to confirm these associations. AdaBoost proved to be the most accurate algorithm for predicting daily AOM visits, as confirmed by its strong validation performance. The SHAP analysis highlighted mean temperature as the dominant predictive variable (contributing 44% to model output), with PM_10_, NO₂, and O₃ exerting smaller yet meaningful influences. The DLNM model confirmed a consistent and statistically significant effect of temperature and moderate delayed effects for NO₂ and PM_10_ alongside minor but significant short-lag effects for O₃. The integrated approach, based on the combined use of Machine Learning and models capable of estimating short-term delayed (dose-response) effects, proposed in this study, represents a methodological contribution for a deeper epidemiological understanding of the timing and intensity of environmental exposure effects. The complexity of pathogenetic mechanisms in the onset of acute diseases and the interaction between multiple environmental factors constitute a challenge that can benefit from new methodologies to find effective health prevention strategies for diseases associated with adverse environmental conditions.

## Data Availability

The data supporting the findings of this study were provided by S. Croce and Carle Hospital, Cuneo, Italy (Emergency Department records, 2007–2015). Due to privacy and ethical restrictions, the original datasets are not publicly available. However, statistical summaries are reported within the article. Researchers interested in accessing the data may contact the corresponding author for reasonable requests, subject to approval by S. Croce and Carle Hospital.
